# Photoinduced Reversible Bending and Guest Molecule Release of Azobenzene-Containing Polydiacetylene Nanotubes

**DOI:** 10.1038/s41598-019-52462-5

**Published:** 2019-11-05

**Authors:** Daewoong Jang, Sumit Kumar Pramanik, Amitava Das, Woohyun Baek, Jung-Moo Heo, Hyun-Joo Ro, Sangmi Jun, Bum Jun Park, Jong-Man Kim

**Affiliations:** 1 Department of Chemical Engineering, Hanyang University, Seoul, 04763 Korea; 2 CSIR-Central Salt & Marine Chemicals Research Institute, Bhavnagar, 364002 Gujarat India; 3 Drug and Disease Target Team, Korea Basic Science Institute, Cheongu, 28119 Korea; 4 Convergent Research Center for Emerging Virus Infection Korea Research Institute of Chemical Technology, Daejeon, 34114 Korea; 5 Department of Chemical Engineering, Kyung Hee University, Yongin, 17104 Korea; 6 Institute of Nano Science and Technology, Hanyang University, Seoul, 04763 Korea

**Keywords:** Chemistry, Materials science

## Abstract

Creation of hollow, one-dimensional nanomaterials has gained great recent attention in the chemical and material sciences. In a study aimed at discovering new functional materials of this type, we observed that an amphiphilic diacetylene (DA) derivative, containing an azobenzene moiety and an oligo-ethylene group, self-assembles to form nanotubes and undergoes photopolymerization to form hollow polydiacetylene (PDA) nanotubes with a uniform wall thickness and diameter. The azobenzene-PDA nanotubes are photoresponsive in that on-and-off UV-irradiation leads to a reversible morphological change between straight and bent forms in association with E-Z photoisomerization of the azobenzene group. Owing to the UV-induced structural change feature, the new DA and PDA nanotubes serve as a controlled release material. Accordingly, fluorescent rhodamine B encapsulated inside the nanotubes are effectively released by using repeated on-off UV irradiation. Furthermore, photo-release of rhodamine B was shown to occur in an artemia (brine shrimp).

## Introduction

Polydiacetylenes (PDAs), a family of structurally and optically unique conjugated polymers, can be readily prepared by polymerization (typically 254 nm UV-irradiation, heat treatment or electric pulse) of well-ordered assemblies of diacetylenes (DAs)^1–10^. The PDAs generated by polymerization of the DAs contain extensively π-conjugated ene-yne systems within molecular structures. In addition, PDAs often display blue-to-red color and fluorescence-turn-on changes in response to various external stimuli in association with partial distortions of the π-conjugated backbones^11–14^. These unique optical features enable the use of PDAs as stimulus-responsive colorimetric sensing and imaging materials^15–33^. We also reported PDA-based chromatic sensor systems^34–37^.

Azobenzene compounds possess photo-response properties associated with their ability to undergo reversible isomerization between energetically stable E and unstable Z forms upon exposure to and removal of 365 nm UV light, respectively^38–40^. Because the photochemically-thermally induced reversible isomerization process alters their molecular shapes, azobenzenes have been actively utilized as key components in actuators^41–47^. In addition, owing to the facile π-π stacking interaction, certain azobenzene derivatives form tubular materials when they are subjected to the self-assembly condition^48–53^.

**Figure 1:**
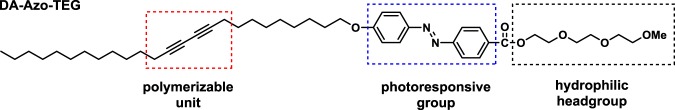
Structure of an azobenzene-containing diacetylene DA-Azo-TEG.

By combining the properties of azobenzenes and PDAs, it has been possible to design azobenzene-containing PDA systems^54–62^ and some of these systems displayed photo-induced colorimetric changes^61,62^. We also reported that an azobenzene-containing diacetylene monomer forms a crystalline, blue-colored PDA upon irradiation with 254 nm light^62^. Interestingly, this azobenzene-linked supramolecular PDA undergoes repeatable and a reversible blue-to-red color change and crystal tearing upon being irradiated with 330–380 nm UV light. These phenomena are attributed to large structural changes in the PDA backbone created by E-Z isomerization of the azobenzene moiety. Very interestingly, introduction of a hydrophilic triethylene glycol (TEG) moiety to an azobenzene-containing DA molecule resulted in the generation of completely different morphologies. Thus, the amphiphilic **DA-Azo-TEG** (Fig. 1) afforded tubular supramolecules when it was subjected to a self-assembly condition (aqueous ethanol). Polymerized **PDA-Azo-TEG** nanotubes were readily prepared by 254 nm UV irradiation owing to the presence of the photopolymerizable DA unit in the middle of the hydrophobic alkyl chain. Additional meritorious feature of the DA unit is that the resultant PDA nanotube is chemically and mechanically robust. Note that the majority of the organic nanotubes formed via self-assembly techniques loses their periodic regularities upon exposure to organic solvents that is frequently required for processing, because the driving forces for self-assembling molecular building blocks are typically on the basis of hydrogen bonds, aromatic interactions, anti-solvent effects, and van der Waals forces^50,63–67^. The hydrophilic TEG group is believed to play a critical role for the formation of the nanotube in an aqueous ethanol solution. The photoresponsive azobenzene moiety was introduced to induce bending motion of the resultant nanotube. Combination of the photopolymerizable DA unit, an azobenzene group and the hydrophilic TEG afforded fabrication of responsive nanotubes with bending vibration and guest molecule releasing properties.

## Results and Discussion

### Fabrication and analysis of PDA-Azo-TEG nanotube

**Figure 2:**
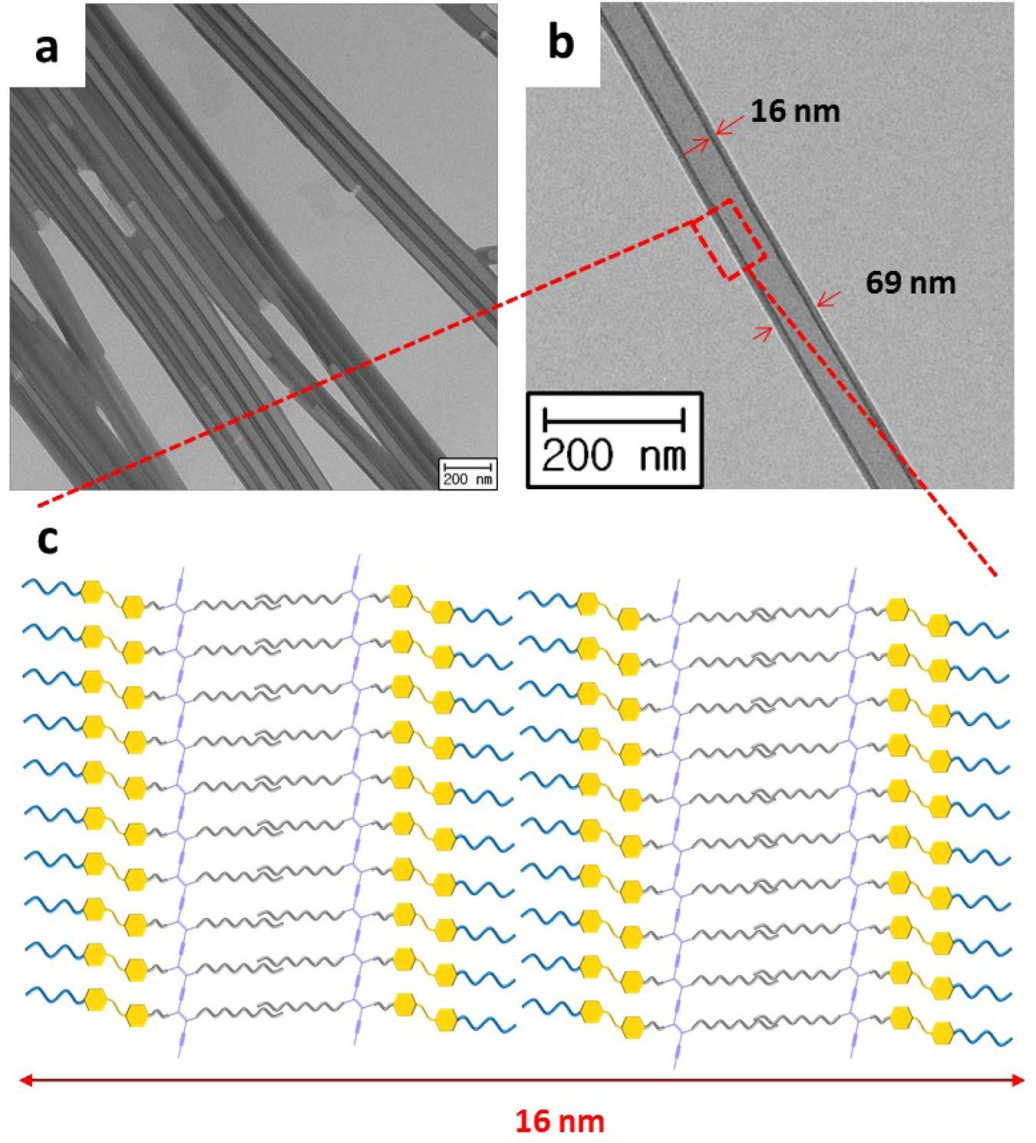
Morphologies of PDA-Azo-TEG nanotubes. (**a**,**b**) TEM images showing hollow nanotubes. (**c**) Schematic illustrating the multilayered nanotube wall.

A hot aqueous ethanol solution (1:1 (v/v) H_2_O:EtOH) of **DA-Azo-TEG** became turbid upon cooling at −7 °C for 48 h and a light yellow precipitate formed (yield > 80%). The light yellow precipitate was photopolymerized by 254 nm UV irradiation, resulting in a blue color, which is a typical characteristic of self-assembled DAs. Interestingly, TEM analysis of the precipitate revealed the existence of tubular structures (Fig. 2a), having uniform outer diameters of *ca*. 69 nm and thicknesses of *ca*. 16 nm (Fig. 2b). The XRD diffraction pattern of the **PDA-Azo-TEG** nanotube powder demonstrated that this material exists as a multilayered self-assembly structure of nanotubes (Fig. S1). The strongest intensity peak in the XRD pattern at 2θ = 2.44° corresponds to a d-spacing of λ = 3.94 nm, the interlamellar distance based on the Bragg’s law. The observations suggest that DA molecules with a length of *ca*. 5.25 nm are assembled laterally in the form of a lamellar structure and that the DA layers are radially and cylindrically stacked resulting in a double bilayer array (Fig. 2c). Note, the tube wall thickness is approximately four-times greater than the interlamellar distance (δ ≈ 4λ). Importantly, the dimensions (i.e., wall thicknesses and diameters) of the **DA-Azo-TEG** are nearly the same as those of **PDA-Azo-TEG**, indicating that polymerization of the DA occurs with little to no change in the shape of the material (Fig. S2).

**Figure 3:**
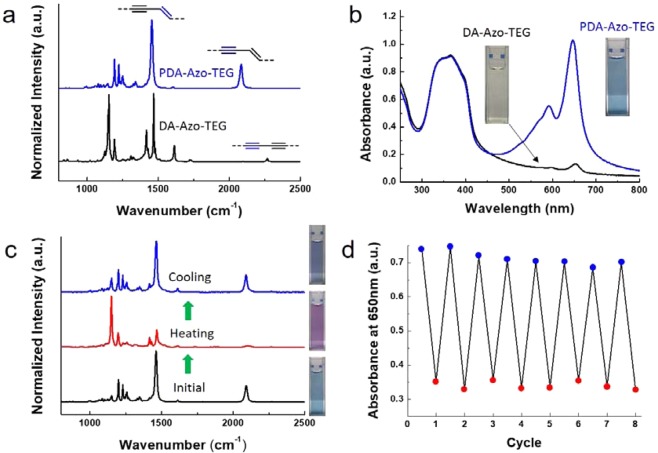
Reversible thermochromic properties of PDA-Azo-TEG nanotubes. (**a**,**b**) Raman (**a**) and UV-Vis absorption spectra (**b**) of nanotubes before (black curve, DA-Azo-TEG) and after (blue curve, PDA-Azo-TEG) 254 nm UV irradiation with intensity of 1 mW/cm^2^ for 10 min. The insets in panel b indicate the photographs of corresponding nanotube dispersions. (**c**) Raman spectra of PDA-Azo-TEG dispersions before heating (black curve), right after heating (red curve) at 90 °C, and after cooling (blue curve) to room temperature. (**d**) Reversible thermochromism of PDA-Azo-TEG coated films over multiple runs of heating to 90 °C for 5 seconds and cooling to room temperature for 5 seconds. The blue and red dots indicate absorbance at 650 nm at the heating and cooling conditions, respectively.

The **DA-Azo-TEG** nanotubes in the powder undergo photopolymerization upon irradiation using 254 nm UV light (1 mW/cm^2^) for 10 min. Raman spectroscopic analysis (Fig. 3a) shows that upon polymerization the peak at 2268 cm^−1^ corresponding to the triple bond in **DA-Azo-TEG** disappears while the characteristic peaks at 2084 and 1454 cm^−1^ that correspond to the triple and double bonds in **PDA-Azo-TEG** arise. It was also observed that **DA-Azo-TEG** nanotubes dispersed in a solution of 1:1 (v/v) ethanol-water display a light yellow color, which changes to blue upon irradiation with 254 nm light. This change is reflected more clearly in the UV-Vis spectra displayed in Fig. 3b, where the strong peak at *ca*. 650 nm associated with the characteristic blue phase of π-conjugated PDAs arises as the dispersion of **DA-Azo-TEG** is exposed to UV light. The absorbance at 650 nm of the **DA-Azo-TEG** dispersion increased over irradiation time, and the growth rate of absorbance gradually decreased (Fig. S3). When gradually heated from ambient temperature to 90 °C, the color change from blue to red was observed in the **PDA-Azo-TEG** nanotube dispersion (Fig. 3c). A reverse color change from red to blue occurs when the dispersion is cooled to room temperature. In a manner that is consistent with the blue-to-red color transition, the intensities of Raman peaks at 2090 and 1464 cm^−1^, corresponding to the respective conjugated triple and double bonds in **PDA-Azo-TEG**, decrease and their positions shift slightly to 2104 and 1467 cm^−1^, respectively, when the blue phase dispersion is heated^68^. Furthermore, the thermochromic transitions of **PDA-Azo-TEG** nanotubes, as monitored by absorbances at 650 nm (Fig. 3d), occur reversibly over several consecutive heating-cooling cycles. In addition, the integrity of the tubular morphology of **PDA-Azo-TEG** is significantly greater than that of DA-Azo-TEG. Specifically, we observed that the tubular shape is lost when the dried DA-Azo-TEG nanotubes are exposed to a few drops of organic solvents (Fig. S4a), whereas the tubular morphology of **PDA-Azo-TEG** is maintained under these conditions (Fig. S4b).

### Photoinduced bending motion of nanotube

Perhaps the most intriguing property of **PDA-Azo-TEG** is its ability to undergo morphological changes when exposed to UV-light, which causes E-to-Z isomerization of the azobenzene moiety. For example, **PDA-Azo-TEG** nanotubes experience reversible stretching and bending when subjected to on-and-off 365 nm UV irradiation. For a more accurate assessment of these changes, TEM analysis was carried out on three, xenon light illuminated samples (a–c), which were prepared by placing a drop of the **PDA-Azo-TEG** nanotube dispersion on TEM grids and followed by drying at room temperature in the (a) absence and (b) presence of 365 nm UV-irradiation, and (c) by placing a 365 nm UV-irradiated nanotube dispersion on a TEM grid followed by drying at room temperature.

**Figure 4:**
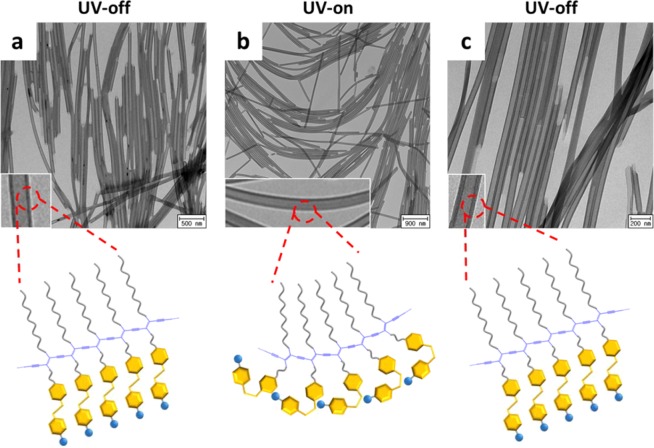
Mechanically reversible photoresponses of PDA-Azo-TEG nanotubes upon on-and-off 365 nm UV-irradiation. TEM images of PDA-Azo-TEG nanotube samples prepared in the conditions of (**a**) UV-off, (**b**) UV-on, and (**c**) UV-off. Insets in each panel are magnified images. Schematics indicate the prospective PDA internal structures over the UV-induced isomerization, where the yellow hexagons and blue circles denote azobenzene and TEG groups, respectively.

As can be seen in Fig. 4a, the TEM image of sample (a) reveals the existence of a bundle of linear nanotubes, whereas that of the nanotubes exposed to UV irradiation during drying (sample (b)) have bent structures (Fig. 4b). Moreover, in the TEM image (Fig. 4c) of the sample generated by 365 nm UV exposure followed drying in the absence of UV irradiation, the **PDA-Azo-TEG** nanotubes have linear tubular structures. Finally, the TEM image of **PDA-Azo-TEG**, pre-exposed to 365 nm UV followed by irradiation with visible light during solvent removal, demonstrates the existence of reversibly formed, straight tubular structures.

It is reasonable to suggest that the changes occurring in the **PDA-Azo-TEG** morphological structures are associated with *E*-*Z* photoisomerization about the N = N bond in the azobenzene moiety, which is schematically illustrated in Fig. 4. Support for this proposal comes from the results of UV-Vis absorption spectroscopic analysis (Fig. S5). Notably, exposure of a dispersion of **DA-Azo-TEG** to 365 nm UV causes a decrease in the absorbance at *ca*. 360 nm and a corresponding increase in the intensities of absorption bands at *ca*. 300 and 450 nm, spectroscopic changes that are typically observed during *E*-to-*Z* photoisomerization of azobenzene compounds^69^. In addition, the intensity of the absorption band at *ca*. 360 nm is recovered when the 365 nm UV irradiation is terminated, revealing that reverse, thermal *Z*-to-*E* isomerization takes place.

### Photoinduced release of guest from PDA-Azo-TEG

As described above, a distinct change occurs in the shape of the **PDA-Azo-TEG** nanotubes from linear to bent upon UV-light irradiation. It was expected that this morphological change would create a mechanical force within the tubes that might be used to drive release of guests encapsulated in the hollow cylindrical nanotubes. This capability was assessed using the fluorescent dye rhodamine B as a model guest. It is important to note that in comparison to its emission in solution, the fluorescence of rhodamine B is partially quenched when it is entrapped inside the nanotubes as a consequence of self-quenching (Fig. S6). Thus, relative fluorescence intensity measurements enabled the evaluation of the relative distribution of nanotube-entrapped versus free rhodamine B in the form of a percentage of released dye relative to the total amount of rhodamine B initially encapsulated within nanotubes.

**Figure 5:**
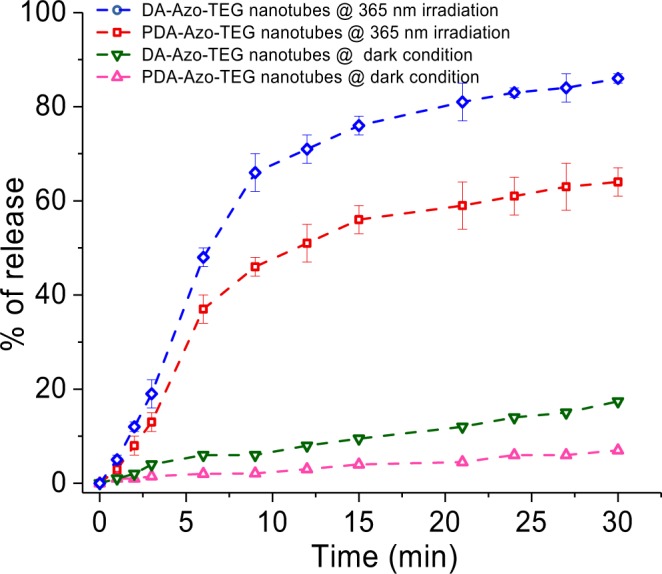
Dye-release profile of encapsulated rhodamine B from DA-Azo-TEG nanotubes exposed at 365 nm UV irradiation (blue line) and kept under dark condition (green line) and PDA-Azo-TEG nanotubes exposed at 365 nm UV irradiation (red line) and kept under dark condition (pink line).

Nanotubes containing encapsulated rhodamine B were prepared by mixing the lyophilized nanotubes (5.0 mg) in a HEPES buffer solution (pH 7.4) containing rhodamine B (50 mg, 0.104 mmoles). Capillary force enabled the nanotubes to encapsulate rhodamine B. After overnight incubation, the solution was filtered through a polycarbonate membrane with 0.2 mm pore size, giving nanotubes that were washed several times with HEPES buffer to remove rhodamine B on the exterior of the nanotubes. Complete destruction of the nanotubes, promoted by addition of 2% Triton X-100, caused total release rhodamine B. The fluorescence intensity at 566 nm of the solution of decomposed nanotubes was set as 100% (maximum fluorescence intensity, F_0_) (Fig. 5). Fluorescence intensities of solutions after release of rhodamine B (F_t_) were then used to determine the % Release (F_t_/F_0_ × 100) shown in Fig. 5.

The results demonstrate that a distinct enhancement in the amount of dye takes place upon UV irradiation of the rhodamine B pre-encapsulated **PDA-Azo-TEG** nanotubes. In the absence of UV irradiation, a gradual but slow release of encapsulated rhodamine B occurs from both dye-loaded **DA-Azo-TEG** and **PDA-Azo-TEG** in pH 7.4 solutions, which is expected for nanotubular arrays that have open ends and cylindrical morphologies with high-axial-ratios^70^. The extent of release of rhodamine B in the dark for a 30 min period is about 5% for the **PDA-Azo-TEG** nanotubes and *ca*. 10% for **DA-Azo-TEG** nanotubes. In contrast, UV irradiation prompts substantial release of encapsulated rhodamine B to the extents of 60% for **PDA-Azo-TEG** nanotube and 84% for **DA-Azo-TEG** nanotube. Increases in the amounts of rhodamine B release were found to match the extents of E-Z isomerization of the azobenzene unit over 30 min periods^71^. The fact that release of encapsulated rhodamine B is not complete under UV-light irradiation conditions indicates that the nanotube shells in the E-forms of **DA-Azo-TEG** and **PDA-Azo-TEG** have cavities, in which rhodamine B is tightly bound^53^.

**Figure 6:**
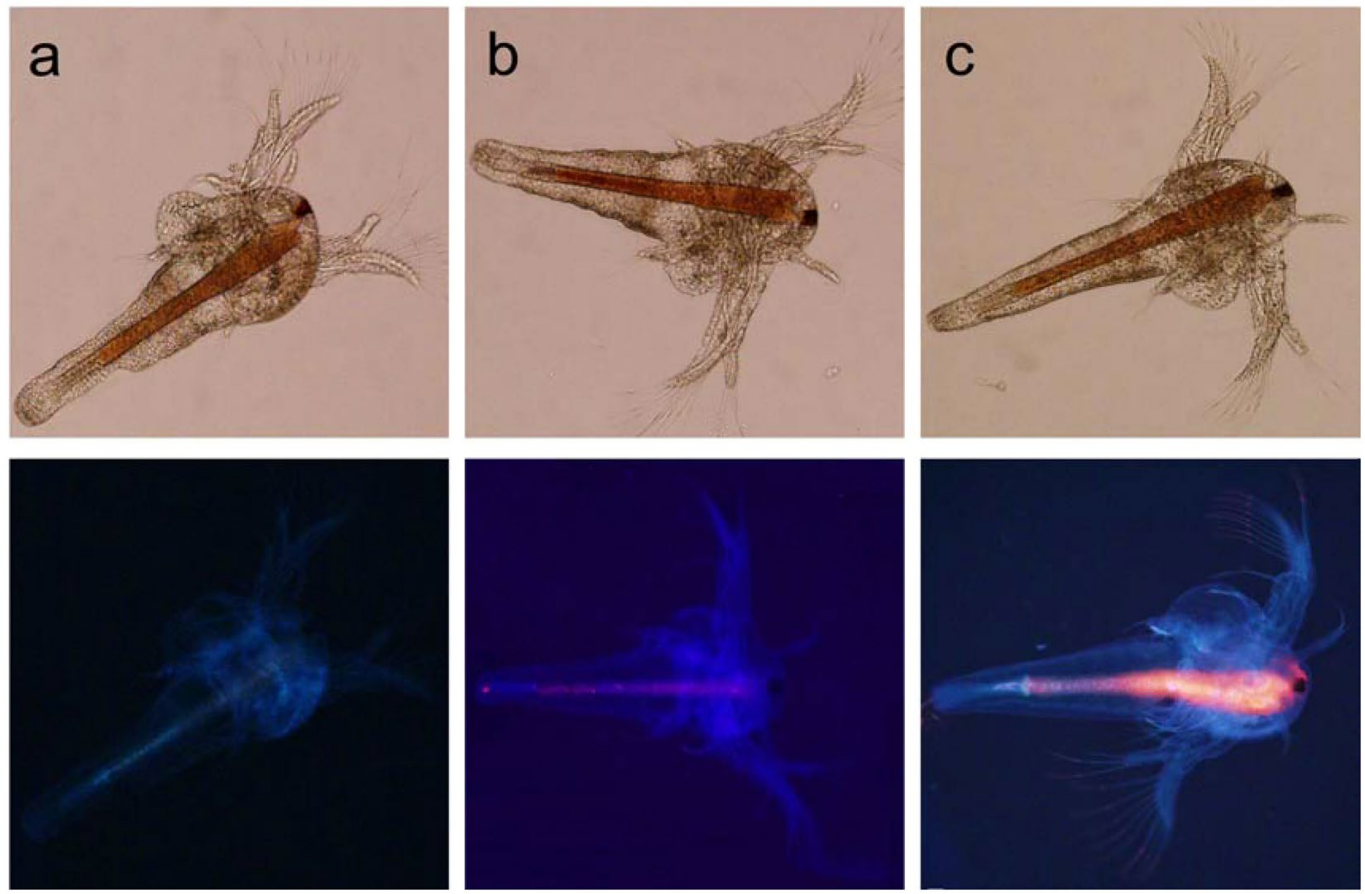
Brightfield & fluorescence images of Artemia after 24 h hatching: (**a**) in pure sea water; (**b**) incubated in rhodamine B encapsulated nanotube dispersion for 20 min (**c**) after 365 nm UV irradiation for 4 minutes (Upper row indicate bright field images; lower row represent the corresponding fluorescence images).

After demonstrating the photo-responsiveness of the polymeric shell of the nanotube and achieving the subsequent controlled release of rhodamine B, experiments with the invertebrate model Artemia (brine-shrimp) (Fig. 6a) were performed to explore the use of nanotubular **DA-Azo-TEG** for controlled photo-triggered release of an encapsulated payload in living systems. Upon incubation of artemia with rhodamine B pre-loaded **DA-Azo-TEG** nanotubes, fluorescence emission can be observed throughout the gastrointestinal tract of the organism (Fig. 6b). Upon photo-irradiation, a significant enhancement in the emission intensity of rhodamine B in treated artemia occurs (Fig. 6c). This observation shows that **DA-Azo-TEG** nanotubes have potential for use in stimuli-responsive, real time release of encapsulated payloads in living systems.

## Conclusion

In the investigation described above, we developed an effective approach for fabricating a photoresponsive PDA nanotube that has a uniform wall thickness and diameter. The DA monomer, employed to generate the PDA nanotubes, contains an azobenzene moiety for photo-triggered shape alteration and TEG groups, which provide it with amphiphilic character that enables spontaneous formation of hollow nanotubes with multilayered lamellar walls structures. The mechanical properties and resistance of the **DA-Azo-TEG** nanotubes was found to be drastically improved upon photopolymerization to form the corresponding PDA nanotubes.

The **PDA-Azo-TEG** nanotubes have a blue color as a result of the presence of highly extended π-conjugation along the polymer backbone. Interestingly, a color change from blue-to-red takes place when the **PDA-Azo-TEG** nanotubes are heated, and the change is reversed upon cooling. Furthermore, the results of this effort show that the **PDA-Azo-TEG** nanotubes undergo a morphological change from linear to bent upon 365 nm UV-irradiation, which promotes *E*-to-*Z* isomerization about the N = N bonds in the azobenzene groups. Importantly, this shape change is reversed when irradiation is terminated as a result of the operation of the well-known azobenzene thermal *Z*-to-*E* isomerization process. Owing to the existence of hollow and cylindrical cores, the **DA-Azo-TEG** and **PDA-Azo-TEG** nanotubes encapsulate the guest molecule, rhodamine B. Moreover, UV-irradiation of both rhodamine B encapsulating nanotubes promotes release of the dye. Finally, the results of this effort show that the photo-triggered dye release from rhodamine-encapsulated D**A-Azo-TEG** also occurs in live brine shrimp.

## Experimental Section

### Materials

10,12-Pentacosadiynoic acid (PCDA) was purchased from GFS Chemicals (Ohio, USA). Lithium aluminum hydride (LiAlH_4_), tetrabromomethane, triphenylphosphine, potassium iodide, and 4-dimethylaminopyridine (DMAP) were purchased from Sigma-Aldrich. N-(3-Dimethylaminopropyl)-N’-ethylcarbodiimide hydrochloride (EDC) was purchased from TCI chemicals (Tokyo, Japan). Potassium carbonate, sodium hydroxide, and sodium nitrite were purchased from Daejung Chemicals & Metals (Seoul, South Korea). Hydrochloric acid and methyl-4-aminobenzoate were purchased from Fisher Scientific (Massachusetts, USA). The detailed synthetic protocols for **DA-Azo-TEG** and spectral data are provided in Supporting Information.

### Instrumentation

^1^H NMR and ^13^C NMR spectra were obtained with a Varian VNMRS (600 MHz) spectrometer. UV-vis spectra were recorded on a single beam Agilent 8453 UV-vis spectrometer (Agilent Technologies). Absorption spectra were obtained with a USB2000 miniature fiber-optic spectrometer (Ocean Optics). XRD spectra were recorded on a D8 discover (Bruker). Optical microscopic images were obtained with an Olympus BX 51 W/DP70 microscope. TEM images were collected using a Zeiss LEO912AB microscope. SEM images were collected with a NOVA NanoSEM 450 at an accelerating voltage of 15 kV. IR spectra were recorded on a Thermo Nicolet NEXUS 870 FT-IR with KBr plate. Raman spectra were obtained with a Raman microscope (785 nm laser, LabRAM HR Evolution, Horiba).

### Fabrication of DA-Azo-TEG and PDA-Azo-TEG nanotubes

A hot (70 °C) ethanol (5 mL) solution of **DA-Azo-TEG** (5 mg) was added to a 5 mL of hot (70 °C) deionized water. The clear solution was placed in a refrigerator (−7 °C) for 48 h. The formed pale yellow precipitate was collected (4.3 mg) by filtration. Photopolymerization of the precipitate with 254 nm UV (1 mW/cm^2^, 5 min) resulted in the generation of **PDA-Azo-TEG** nanotubes, which were subjected to UV-Visible and Raman spectroscopic analyses.

### Photoinduced bending

A suspension (3 mL) of self-assembled **DA-Azo-TEG** in 50% ethanol-water was placed in a Petri dish and irradiated with UV light (365 nm, 25 mW/cm^2^) until the solvent evaporated completely. TEM analysis was carried out with samples obtained both before and after removal of the solvent. In addition, morphologies of the materials were assessed by using TEM after repeated 365 nm UV on-off cycles.

### Photoinduced release of guest molecules

Nanotubes encapsulating rhodamine B (λ_abs max_ = 544 nm, λ_em max_ = 566 nm) were prepared by mixing HEPES buffer solutions of rhodamine B (50 mg, 0.104 mmoles) with the lyophilized **DA-Azo-TEG** and **PDA-Azo-TEG** nanotubes (5.0 mg) at pH 7.4. After overnight incubation, the rhodamine B encapsulating nanotubes were collected by filtration through a 0.2 mm pore polycarbonate membrane and washed several times with HEPES buffer to remove rhodamine B on the exteriors of the nanotubes. Complete destruction of the nanotubes, promoted by addition of 2% Triton X-100, caused total release rhodamine B. The fluorescence intensity at 566 nm of the solution of decomposed nanotubes was set as 100% (maximum fluorescence intensity) in Fig. 5 at (F_0_). The amount of encapsulated rhodamine B in the nanotube (5.0 mg) was estimated to be 1.2 mg. Fluorescence intensities (F_t_) of released rhodamine B were then used to determine the % Release (F_t_/F_0_ $\times \,$100) in Fig. 5. All measurements were performed in triplicate and an average value is given in Fig. 5.

### Photoinduced release of rhodamine B from DA-Azo-TEG nanotubes in artemia

Artemia culture and staining experiments were performed using natural autoclaved seawater and purging with 0.22 µm filtered air. Artemia cysts (1.0 g), purchased from Tetra (Tokyo, Japan), were decapsulated by adding the 200 mL of 30% sodium hydroxide and 50% sodium hypochlorite, followed by adding 15 mL 1% sodium thiosulfate (10 g/L) after 2 min. The decapsulated cysts were resuspended in 500 mL sterile natural seawater and allowed to hatch over a 24 h period at room temperature with vigorous air purging under constant illumination (*ca*. 5000 lx). Healthy nauplii (500) were randomly collected in 500 mL physiological saline for 1 d. Artemia nauplii in physiological saline were incubated with rhodamine B loaded **DA-Azo-TEG** nanotubes (1 mg, 10 mL) for 30 min at 37 °C and exposed to UV light (365 nm, 50 mW/cm^2^) for 10 min. Imaging of Artemia nauplii was performed using a fluorescence microscope with a 20 × microscope objective (Olympus).

## Supplementary information

Supplementary information

## References

[1] Wegner G, Mitt I (1969). Polymerisation von Derivaten des 2.4-Hexadiin-1.6-diols im Kristallinen Zustand. Z. Naturforsch., Teil B.

[2] Tahir MN, Nyayachavadi A, Morin J-F, Rondeau-Gagné S (2018). Recent Progress in the Stabilization of Supramolecular Assemblies with Functional Polydiacetylenes. Polym. Chem..

[3] Yarimaga O, Jaworski J, Yoon B, Kim J-M (2012). Polydiacetylenes: Supramolecular Smart Materials with a Structural Hierarchy for Sensing, Imaging and Display Applications. Chem. Commun..

[4] Jelinek R, Ritenberg M (2013). Polydiacetylenes–Recent Molecular Advances and Applications. RSC Adv..

[5] Okawa Y, Akai-Kasaya M, Kuwahara Y, Mandal SK, Aono M (2012). Controlled Chain Polymerisation and Chemical Soldering for Single-Molecule Electronics. Nanoscale.

[6] Verstraete L, Hirsch BE, Greenwood J, De Feyer S (2017). Confined Polydiacetylene Polymerization Reactions for Programmed Length Control. Chem. Commun..

[7] Song P, Qin H, Gao H-L, Cong H-P, Yu S-H (2018). Self-healing and Superstretchable Conductors from Hierarchical Nanowire Assemblies. Nat. Commun..

[8] Spagnoli S, Briand E, Vickridge I, Fave J-L, Schott M (2017). Method for Determining the Polymer Content in Nonsoluble Polydiacetylene Films: Application to Pentacosadiynoic Acid. Langmuir.

[9] Jiang H, Ehlers M, Hu X-y, Zellermann E, Schmuck C (2018). Dimensional Control of Supramolecular Assemblies of Diacetylene-Derived Peptide Gemini Amphiphile: from Spherical Micelles to Foamlike Networks. Soft Matter.

[10] Fernandez-Castano Romera M (2017). Strain Stiffening Hydrogels through Self-Assembly and Covalent Fixation of Semi-Flexible Fibers. Angew. Chem. Int. Ed..

[11] Sun X, Chen T, Huang S, Li L, Peng H (2010). Chromatic Polydiacetylene with Novel Sensitivity. Chem. Soc. Rev..

[12] Huo J (2017). Advances in Polydiacetylene Development for the Design of Side Chain Groups in Smart Material Applications – a mini review. Polym. Chem..

[13] Chen X, Zhou G, Peng X, Yoon J (2012). Biosensors and Chemosensors Based on the Optical Responses of Polydiacetylenes. Chem. Soc. Rev..

[14] Wen JT, Roper JM, Tsutsui H (2018). Polydiacetylene Supramolecules: Synthesis, Characterization, and Emerging Applications. Ind. Eng. Chem. Res..

[15] Terada H, Imai H, Oaki Y (2018). Visualization and Quantitative Detection of Friction Force by Self‐Organized Organic Layered Composites. Adv. Mater..

[16] Takeuchi M (2018). Tunable Stimuli‐Responsive Color‐Change Properties of Layered Organic Composites. Adv. Funct. Mater..

[17] Lee S (2014). Construction and Molecular Understanding of an Unprecedented, Reversibly Thermochromic Bis‐Polydiacetylene. Adv. Funct. Mater..

[18] Xu Y, Fu S, Liu F, Yu H, Gao J (2018). Multi-Stimuli-Responsiveness of a Novel Polydiacetylene-Based Supramolecular Gel. Soft Matter.

[19] Charych DH, Nagy JO, Spevak W, Bednarski MD (1993). Direct Colorimetric Detection of a Receptor-Ligand Interaction by a Polymerized Bilayer Assembly. Science.

[20] Kolusheva S, Boyer L, Jelinek R (2000). A Colorimetric Assay for Rapid Screening of Antimicrobial Peptides. Nat. Biotech..

[21] Dolai S (2017). Colorimetric Polydiacetylene–Aerogel Detector for Volatile Organic Compounds (VOCs). ACS Appl. Mater. Interfaces.

[22] Wang Y (2017). Synergistic Tailoring of Electrostatic and Hydrophobic Interactions for Rapid and Specific Recognition of Lysophosphatidic Acid, an Early-Stage Ovarian Cancer Biomarker. J. Am. Chem. Soc..

[23] Cui C, Hong NY, Ahn DJ (2018). Monitoring Based on Narrow‐Band Resonance Raman for “Phase‐Shifting” π‐Conjugated Polydiacetylene Vesicles upon Host–Guest Interaction and Thermal Stimuli. Small.

[24] Jiang H (2017). Morphology‐Dependent Cell Imaging by Using a Self‐Assembled Diacetylene Peptide Amphiphile. Angew. Chem. Int. Ed..

[25] Kang DH, Jung H-S, Kim K, Kim J (2017). Mussel-Inspired Universal Bioconjugation of Polydiacetylene Liposome for Droplet-Array Biosensors. ACS Appl. Mater. Interfaces.

[26] Li S, Zhang L, Jiang J, Meng Y, Liu M (2017). Self-Assembled Polydiacetylene Vesicle and Helix with Chiral Interface for Visualized Enantioselective Recognition of Sulfinamide. ACS Appl. Mater. Interfaces..

[27] Meng Y, Jiang J, Liu M (2017). Self-Assembled Nanohelix from a Bolaamphiphilic Diacetylene via Hydrogelation and Selective Responsiveness Towards Amino Acids and Nucleobases. Nanoscale.

[28] Cho Y-S, Ma DH, Ahn KH (2016). Shape-Selective, Stoichiometric Sensing of Fatty Acids with a Mixed Polydiacetylene Liposome. J. Mater. Chem. C.

[29] Seo H, Singha S, Ahn KH (2017). Ratiometric Fluorescence Detection of Anthrax Biomarker with Eu^III^‐EDTA Functionalized Mixed Poly(diacetylene) Liposomes. Asian J. Org. Chem..

[30] Li Z (2018). Point-and-Shoot Strategy for Identification of Alcoholic Beverages. Anal. Chem..

[31] Wang D-E (2018). Polydiacetylene Liposomes with Phenylboronic Acid Tags: a Fluorescence Turn-On Sensor for Sialic Acid Detection and Cell-Surface Glycan Imaging. Nanoscale.

[32] Doerflinger A (2018). Biotin-Functionalized Targeted Polydiacetylene Micelles. Chem. Commun..

[33] Kim S (2017). A Polydiacetylene-Based Colorimetric Chemosensor for Malondialdehyde Detection: a Food Spoilage Indicator. J. Mater. Chem. C.

[34] Park D-H, Hong J, Park IS, Lee CW, Kim J-M (2014). A Colorimetric Hydrocarbon Sensor Employing a Swelling‐Induced Mechanochromic Polydiacetylene. Adv. Funct. Mater..

[35] Chae S, Lee JP, Kim J-M (2016). Mechanically Drawable Thermochromic and Mechanothermochromic Polydiacetylene Sensors. Adv. Funct. Mater..

[36] Lee J (2014). Hydrochromic Conjugated Polymers for Human Sweat Pore Mapping. Nat. Commun..

[37] Lee J (2013). A Protective Layer Approach to Solvatochromic Sensors. Nat. Commun..

[38] Dugave C, Demange L (2003). Cis−Trans Isomerization of Organic Molecules and Biomolecules:  Implications and Applications. Chem. Rev..

[39] Kumar GS, Neckers DC (1989). Photochemistry of Azobenzene-Containing Polymers. Chem. Rev..

[40] Seki T (2018). A Wide Array of Photoinduced Motions in Molecular and Macromolecular Assemblies at Interfaces. Bull. Chem. Soc. Jpn..

[41] Iamsaard S (2016). Fluorinated Azobenzenes for Shape‐Persistent Liquid Crystal Polymer Networks. Angew. Chem. Int. Ed..

[42] Lee J, Oh S, Pyo J, Kim J-M, Je JH (2015). A Light-Driven Supramolecular Nanowire Actuator. Nanoscale.

[43] Lu X, Guo S, Tong X, Xia H, Zhao Y (2017). Tunable Photocontrolled Motions Using Stored Strain Energy in Malleable Azobenzene Liquid Crystalline Polymer Actuators. Adv. Mater..

[44] van Oosten CL, Bastiannsen CWM, Broer DJ (2009). Printed Artificial Cilia from Liquid-Crystal Network Actuators Modularly Driven by Light. Nat. Mater..

[45] Qin C (2017). Tetracarboxylated Azobenzene/Polymer Supramolecular Assemblies as High-Performance Multiresponsive Actuators. ACS Appl. Mater. Interfaces.

[46] Yamada M (2008). Photomobile Polymer Materials: Towards Light‐Driven Plastic Motors. Angew. Chem. Int. Ed..

[47] Nie J, Liu X, Yan Y, Zhang H (2017). Supramolecular Hydrogen-Bonded Photodriven Actuators Based on an Azobenzene-Containing Main-Chain Liquid Crystalline Poly(ester-amide). J. Mater. Chem. C.

[48] Sun H-L, Chen Y, Zhao J, Liu Y (2015). Photocontrolled Reversible Conversion of Nanotube and Nanoparticle Mediated by β‐Cyclodextrin Dimers. Angew. Chem. Int. Ed..

[49] Cao H, Jiang J, Zhu X, Duan P, Liu M (2011). Hierarchical Co-Assembly of Chiral Lipid Nanotubes with an Azobenzene Derivative: Optical and Chiroptical Switching. Soft Matter.

[50] Zhang Y (2017). Bilayers Directly Scrolling up to Form Nanotubes via Self-Assembly of an Achiral Small Molecule. Nanoscale.

[51] Kameta N, Masuda M, Shimizu T (2015). Photoinduced Morphological Transformations of Soft Nanotubes. Chem. Eur. J..

[52] Kameta N (2011). Photoresponsive Soft Nanotubes for Controlled Guest Release. Chem. Eur. J..

[53] Hu Q (2012). Photoresponsive Chiral Nanotubes of Achiral Amphiphilic Azobenzene. Soft Matter.

[54] Kim D-Y, Lee S-A, Jung D, Jeong K-U (2016). Photochemical Isomerization and Topochemical Polymerization of the Programmed Asymmetric Amphiphiles. Sci. Rep..

[55] Kim D-Y, Lee S-A, Park M, Jeong K-U (2015). Dual Photo‐Functionalized Amphiphile for Photo‐Reversible Liquid Crystal Alignments. Chem. Eur. J..

[56] Wang J, Yang G, Jiang H, Zou G, Zhang Q (2013). Photo-Responsive Cholesterol-Substituted Diacetylenic Organogels: Morphology Tuning, Photo-Switching and Photo-Polymerization. Soft Matter.

[57] Hu W, Hao J, Li J, Zou G, Zhang Q (2012). Novel Chromatic Transitions of Azobenzene‐Functionalized Polydiacetylene Aggregates in 1,2‐Dichlorobenzene Solution. Macromol. Chem. Phys..

[58] Jiang H (2011). Control of Supramolecular Chirality for Polydiacetylene LB Films with the Command Azobenzene Derivative Monolayer. J. Mater. Chem..

[59] Zhang X-m (2012). Photo-Induced Polymerization and Isomerization on the Surface Observed by Scanning Tunneling Microscopy. J. Phys. Chm. C.

[60] Wu S, Zhang Q, Bubeck C (2010). Solvent Effects on Structure, Morphology, and Photophysical Properties of an Azo Chromophore-Functionalized Polydiacetylene. Macromolecules.

[61] Sun X (2010). UV-Induced Chromatism of Polydiacetylenic Assemblies. J. Phys. Chem. B.

[62] Baek W (2016). Photoinduced Reversible Phase Transition of Azobenzene-Containing Polydiacetylene Crystals. Chem. Commun..

[63] Hatton FL, Chambon P, McDonald TO, Owen A, Rannard SP (2014). Hyperbranched Polydendrons: a New Controlled Macromolecular Architecture with Self-Assembly in Water and Organic Solvents. Chem. Sci..

[64] John G, Jung JH, Masuda M, Shimizu T (2004). Unsaturation Effect on Gelation Behavior of Aryl Glycolipids. Langmuir.

[65] Shimizu T, Masuda M, Minamikawa H (2005). Supramolecular Nanotube Architectures Based on Amphiphilic Molecules. Chem. Rev..

[66] Stendahl JC, Rao MS, Guler MO, Stupp SI (2006). Intermolecular Forces in the Self‐Assembly of Peptide Amphiphile Nanofibers. Adv. Funct. Mater..

[67] Xu J-F (2014). Hydrogen Bonding Directed Self-Assembly of Small-Molecule Amphiphiles in Water. Org. Lett..

[68] Giorgetti E (2006). UV Polymerization of Self-Assembled Monolayers of a Novel Diacetylene on Silver:  A Spectroscopic Analysis by Surface Plasmon Resonance and Surface Enhanced Raman Scattering. Langmuir.

[69] Yuan T, Dong J, Han G, Wnag G (2016). Polymer Nanoparticles Self-Assembled from Photo-, pH- and Thermo-Responsive Azobenzene-Functionalized PDMAEMA. RSC Adv..

[70] Kameta N, Minamikawa H, Masuda M, Mizuno G, Shimizu T (2008). Controllable Biomolecule Release from Self-Assembled Organic Nanotubes with Asymmetric Surfaces: pH and Temperature Dependence. Soft Matter.

[71] Li Z (2017). Photo-Isomerization and Light-Modulated Aggregation Behavior of Azobenzene-Based Ionic Liquids in Aqueous Solutions. RSC Adv..

