# Supplementary material for: Photoinduced Reversible Bending and Guest Molecule Release of Azobenzene-Containing Polydiacetylene Nanotubes
Source: Sci Rep. 2019 Nov 5;9:15982. doi: 10.1038/s41598-019-52462-5 (PMC6831582; doi:10.1038/s41598-019-52462-5)
Supplement: Supplementary file 1 — Supplementary information [file 41598_2019_52462_MOESM1_ESM.docx]

**Supplementary Information**

**Photoinduced Reversible Bending and Guest Molecule Release of Azobenzene-Containing Polydiacetylene Nanotubes**

Daewoong Jang^1^, Sumit Kumar Pramanik^2^,† Amitava Das^2^*, Woohyun Baek^1^, Jung-Moo Heo^1^, Hyun-Joo Ro^3^, Sangmi Jun^3,4^, Bum Jun Park^5*^, Jong-Man Kim^1,6*^

^1^Department of Chemical Engineering, Hanyang University, Seoul 04763, Korea.

^2^CSIR-Central Salt & Marine Chemicals Research Institute, Bhavnagar 364002, Gujarat, India.

^3^Drug and Disease Target Team, Korea Basic Science Institute, Cheongu 28119, Korea.

^4^Convergent Research Center for Emerging Virus Infection Korea Research Institute of Chemical Technology, Daejeon 34114, Korea.

^5^Department of Chemical Engineering, Kyung Hee University, Yongin 17104, Korea.

^6^Institute of Nano Science and Technology, Hanyang University, Seoul 04763, Korea

**J.-M. Kim: jmk@hanyang.ac.kr; B. J. Park: bjpark@khu.ac.kr, A. Das: a.das@csmri.res.in*

**Scheme S1.** Synthesis procedure of **DA-Azo-TEG** monomer. The intermediates, 1−3, were synthesized according to the literature methods^1,2^.

**Synthesis of (E)-methyl 4-((4-(hepta-2,4-diynyloxy)phenyl)diazenyl)benzoate (4).**

**2** (1.5 g, 3.54 mmol, 0.9 equiv.), potassium carbonate (0.82 g, 5.9 mmol, 1.5 equiv.) and potassium iodide (65 mg, 0.39 mmol 0.1 equiv.) were added to the solution of **3** (1 g, 3.93 mmol, 1 equiv.) in DMF (40 mL) at room temperature under N_2_ atmosphere. The solution mixture was stirred at 60 °C overnight. The crude reactant mixture was diluted with chloroform and washed with NaHCO_3_ (2 × 200 mL), water (2 × 200 mL), and brine (100 mL). The organic layer was dried over anhydrous MgSO_4_ and concentrated under reduced pressure. The residue was subjected to a silica gel column chromatography using chloroform as an eluent to yield **4** (1.65 g, 78 %) as an orange solid. ^1^H NMR (600 MHz, CDCl_3_) : δ 8.18 (s, J = 9 Hz, 2H), 7.93 (m, J = 9 Hz, 2H), 7.90 (m, J = 9Hz, 2H), 7.01 (m, J = 9 Hz, 2H), 4.05 (t, J = 6 Hz, 2H), 3.95 (s, 3H), 2.24 (q, 7.2 Hz, 4H), 1.82 (quint, J = 6.6 Hz, 2H), 1.55~1.25 (m, 32H), 0.87 (t, J = 7.2 Hz, 3H); ^13^C NMR (150 MHz, CDCl_3_) : δ 166.6, 162.3, 155.4, 146.9, 131.1, 130.6, 125.2, 122.3, 114.8, 77.6, 77.5, 68.4, 65.3, 65.2, 52.3, 31.9, 29.64, 29.62, 29.60, 29.47, 29.35, 29.34, 29.28, 29.14, 29.09, 28.99, 28.85, 28.78, 28.35, 28.31, 26.0, 22.6, 19.20, 19.19, 14.1; IR (KBr) vcm^-1^ : 2937, 2919, 2849, 1724, 1717, 1602, 1584, 1500, 1477, 1467, 1439, 1419, 1395, 1283, 1247, 1145, 1114, 1038, 1012, 867, 837; MS(MALDI-TOF, m/z) : calcd. for C_39_H_54_N_2_O_3_ [M+H]^+^ 599.4, found 599.38.

**Synthesis of (E)-4-((4-(pentacosa-10,12-diynyloxy)phenyl)diazenyl)benzoic acid (5)**

NaOH (0.33 g, 8.26 mmol, 3 equiv.) dissolved in H_2_O (20 mL) and MeOH (10 mL) was added to the solution of **4** (1.65 g, 2.76 mmol, 1 equiv.) in THF (40 mL) at ambient temperature under N_2_ environment. The solution mixture was stirred for 24 h. The organic solvent of the crude reactant mixture was concentrated under reduced pressure, diluted with water, and acidified with 1 M HCl aqueous solution. The precipitate was washed with water and dried in vacuo to afford **5** (1.5 g, 93%) as a pale yellow solid. ^1^H NMR (600 MHz, THF-d_8_) : δ 8.33 (d, J = 8.4 Hz, 2H), 8.11 (d, J = 9 Hz, 2H), 8.08 (d, J = 9 Hz, 2H), 7.24 (d, J = 9 Hz, 2H), 7.04 (t, J = 8.4 Hz, cis), 6.95 (d, J = 9 Hz, cis), 4.26 (t, J = 6.6 Hz, 2H), 4.09 (t, J = 6.6 Hz, cis), 2.41 (q, J = 7.2 Hz, 4H), 2.00 (quint, J = 7.2 Hz, 2H), 1.79 ~ 1.47 (m, 32H), 1.07 (t, J = 7.2 Hz, 3H); 13C NMR (150 MHz, THF-d_8_) : δ 166.1, 162.5, 155.1, 146.8, 139.3, 130.47, 130.43, 124.9, 123.6, 122.0, 118.9, 114.6, 113.9, 76.5, 76.4, 68.1, 66.9, 65.4, 31.8, 29.58, 29.56, 29.54, 29.44, 29.36, 29.27, 29.26, 29.12, 29.03, 28.96, 28.73, 28.69, 28.38, 28.36, 25.9, 24.8, 22.5, 18.5, 13.4; IR (KBr) vcm^-1^ : 3425, 2919, 2849, 1680, 1602, 1585, 1540, 1501, 1467, 1420, 1393, 1304, 1249, 1145, 1104, 1037, 1018, 869, 837, 778, 721; MS(MALDI-TOF, m/z) : calcd. for C_38_H_52_N_2_O_3_ [M+H]^+^ 585.40, found 585.58

**Synthesis of (E)-2-(2-(2-methoxyethoxy)ethoxy)ethyl 4-((4-(pentacosa-10,12-diynyloxy)phenyl)diazenyl) benzoate (DA-Azo-TEG)**

Triethylene glycol monomethyl ether (0.63 g, 3.85 mmol, 1.5 equiv.), N-(3-dimethylaminopropyl)-N’-ethylcarbodiimide hydrochloride (EDC) (0.73 g, 3.85 mmol, 1.5 equiv.), and 4-dimethylaminopyridine (DMAP) (0.47 g, 3.85 mmol, 1.5 equiv.) were added to the solution of **5** (1.50 g, 2.56 mmol, 1 equiv.) in THF (50 mL) at room temperature under N_2_ atmosphere. The solution mixture was stirred for 24 h. The resulting reactant solution was concentrated under reduced pressure and subjected to a silica gel column chromatography using hexane/ethyl acetate (v/v = 4/1) as an eluent to yield **DA-Azo-TEG** (1.35 g, 72%) as an orange solid. ^1^H NMR (600 MHz, CDCl_3_) : δ 8.19 (d, J = 9 Hz, 2H), 7.94 (d, J = 9 Hz, 2H), 7.90 (d, J = 9.6 Hz, 2H), 7.01 (d, J = 9 Hz, 2H), 4.51 (m, 2H), 4.05 (t, J = 6 Hz, 2H), 3.86 (m, 2H), 3.69 (m, 2H), 3.64 (m, 2H), 3.54 (m, 2H), 3.37 (s, 3H), 2.24 (q, J = 7.2 Hz, 4H), 1.82 (m, 2H), 1.54 ~ 1.24 (m, 32H), 0.88 (t, J = 7.2 Hz, 3H) ^13^C NMR (150 MHz, CDCl_3_) : δ 166.1, 162.3, 155.5, 146.9, 131.1, 130.7, 125.2, 122.3, 114.8, 77.6, 77.5, 71.9, 70.74, 70.67, 70.62, 69.2, 68.4, 65.3, 65.2, 64.3, 59.0, 31.9, 29.63, 29.61, 29.59, 29.46, 29.34, 29.33, 29.28, 29.14, 29.08, 28.98, 28.85, 28.78, 28.34, 28.30, 26.0, 22.7, 19.20, 19.19, 14.1 ; IR (KBr) vcm-1 : 2920, 2847, 2360, 1710, 1601, 1579, 1501, 1462, 1418, 1386, 1271, 1249, 1140, 1112, 1070, 1031, 885, 846, 723; MS(MALDI-TOF, m/z) : calcd. for C_45_H_66_N_2_O_6_ [M+H]^+^ 731.49, found 731.64.

**Fabrication of DA-Azo-TEG nanotubes**

**DA-Azo-TEG** (5 mg) was dissolved in 5 mL of 70 °C ethanol. 5 mL of di-water was added to the solution and the mixture was heated to 70 °C. The mixture was then cooled at −7 °C for 48 h.

**Preparation of PDA-Azo-TEG nanotube films**

A piece of glass slides was soaked in DA-Azo-TEG dispersion (5 mL) and dried. 254 nm UV (1 mW/cm^2^) was irradiated for ~5 min.

**Preparation of rhodamine B encapsulated Nanotubes**

Nanotubes encapsulating rhodamine B was done by mixing an HEPES buffer solution of rhodamine B (1 mg) with the lyophilized nanotubes (10.0 mg) at pH 7.4. Capillary action enabled the nanotubes to encapsulate rhodamine B. After socking overnight, the solution was filtered through a polycarbonate membrane with 0.2 mm pore size. The residual nanotubes were washed several times with HEPES buffer to remove rhodamine B outside of the nanotubes. The complete destruction of the nanotubes by addition of 2% Triton X-100 caused a fluorescence recovery of rhodamine B, to release the encapsulated rhodamine B. The fluorescence intensity signal of the of this solution was set as 100% (maximum fluorescence intensity at 520 nm: F_0_). The amount of encapsulated rhodamine B in the nanotube (0.5 mg). The fluorescence intensity, F_t_, of released rhodamine B after a certain time, with and without photoirradiation, was monitored. The measurement was done in triplicate, and an average value was taken. The percentage of rhodamine B release caused by photoirradiation was evaluated by using the following equation:

$$\% of release=\frac{F_{t}}{F_{o}}\times100$$

**
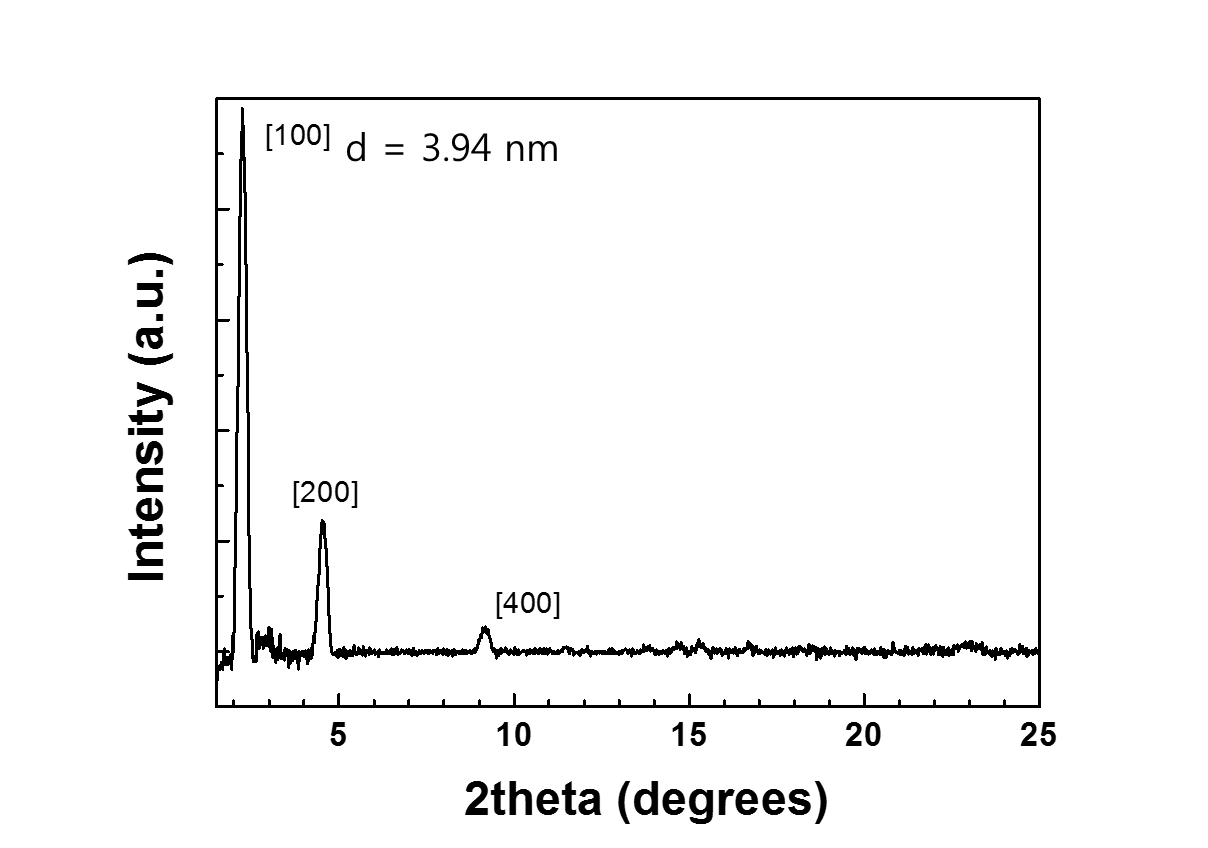
Supplementary Figure S1.** XRD diffraction pattern of **PDA-Azo-TEG** nanotubes.

Supplementary Figure S2. TEM images of (a) DA-Azo-TEG and (b) PDA-Azo-TEG nanotubes. Polymerization was induced by 254nm UV irradiation (1 mW/cm^2^) for 5 min.

**Supplementary Figure S3.** Time evolution of UV absorption spectra of **DA-Azo-TEG** dispersion (a) upon irradiating 254 nm UV (1mW/cm^2^) and (b) the corresponding absorbance at 650 nm as a function of irradiation time._
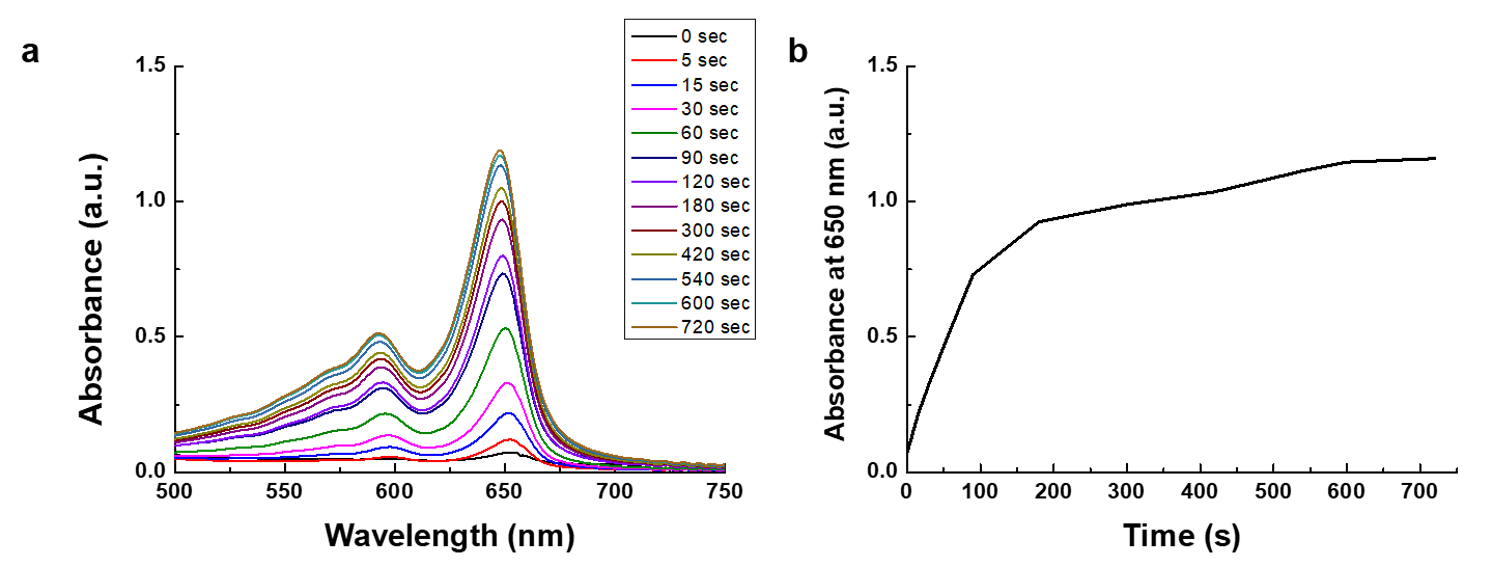
_


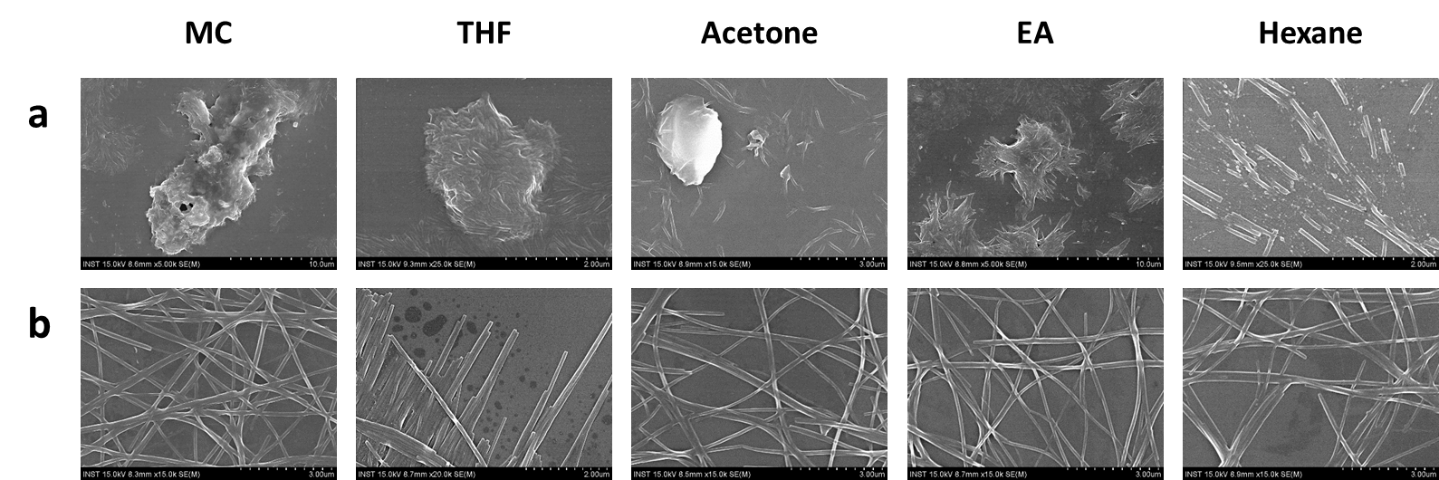


Supplementary Figure S4. Solvent resistance of nanotubes. SEM images of (a) DA-Azo-TEG and (b) PDA-Azo-TEG nanotubes after exposure to various solvents. Polymerization was induced by 254nm UV irradiation (1 mW/cm^2^) for 10 min.


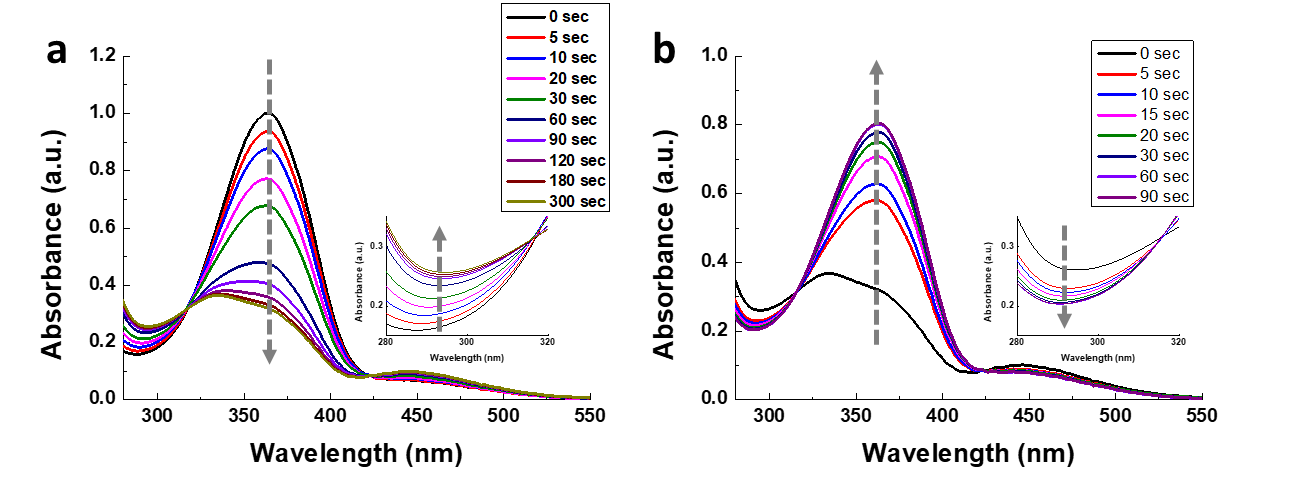


Supplementary Figure S5. Time evolution of UV absorption spectra of DA-Azo-TEG solution (a) upon irradiating 365 nm UV (1mW/cm^2^) over time and (b) upon subsequent exposure of visible light to the DA-Azo-TEG sample solution that was 365 nm UV-irradiated for 300 sec.


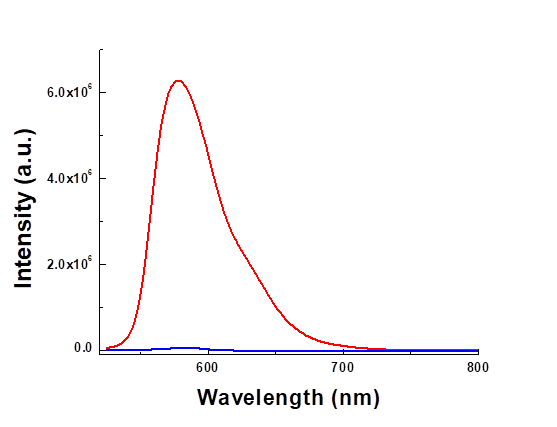
**Supplementary Figure S6**. Fluorescence spectra of **PDA-Azo-TEG** nanotubes with released rhodamine B(red color) and encapsulated rhodamine B(blue color).


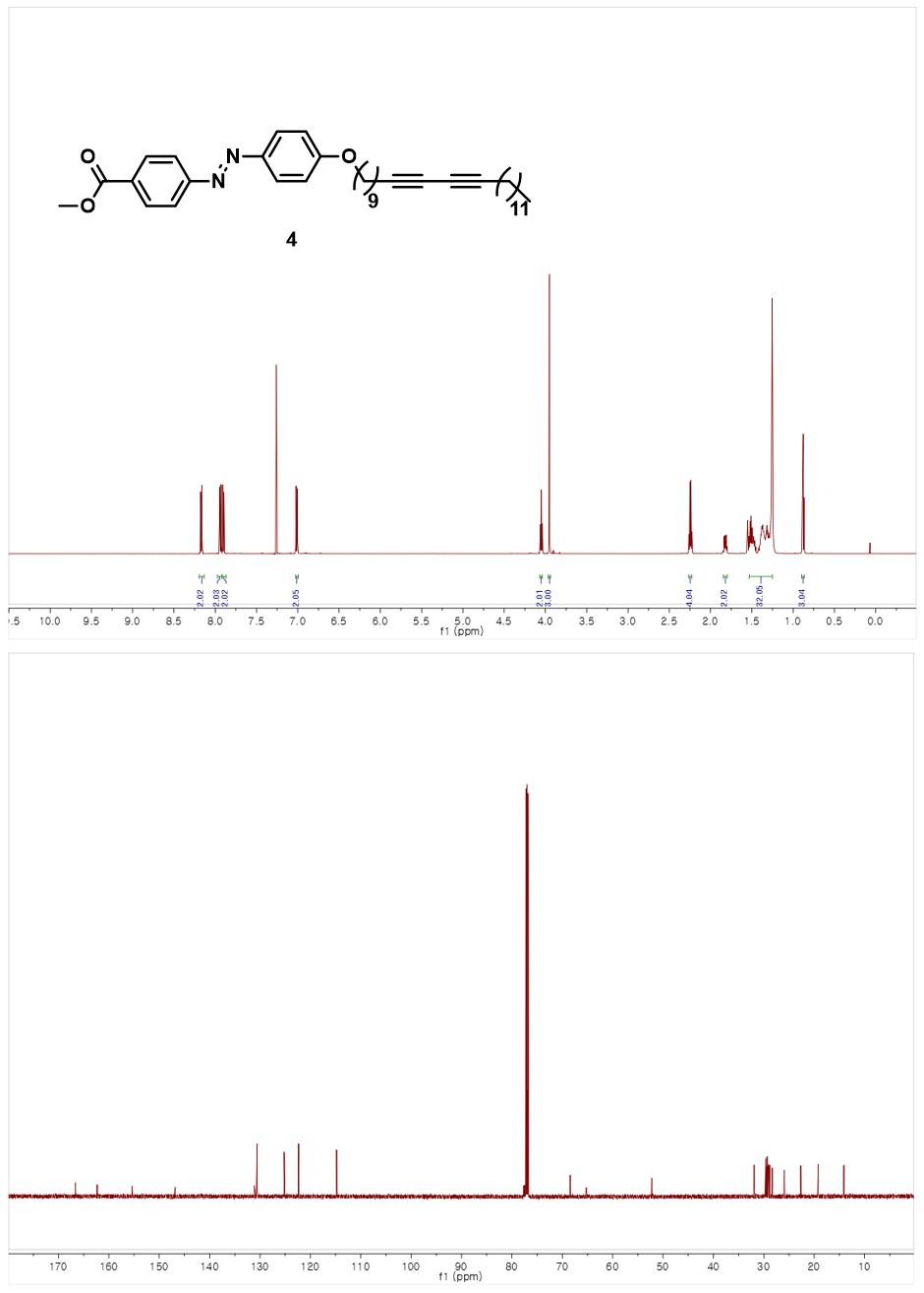


**Supplementary Figure S7.** ^1^H (top, 600 MHz) and ^13^C (bottom, 150 MHz) NMR spectra of **4** in CDCl_3_.


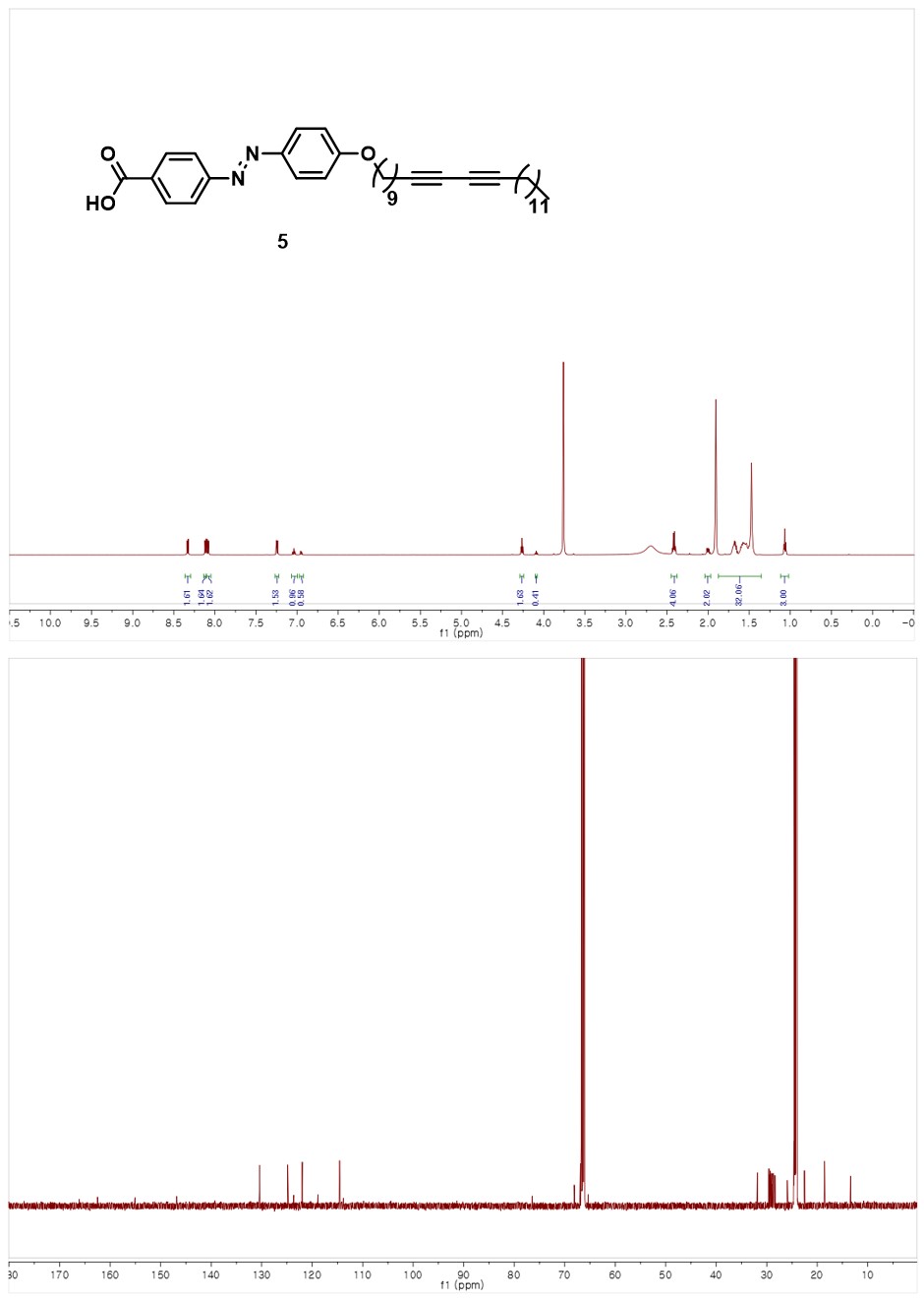


Supplementary Figure S8. ^1^H (top, 600 MHz) and ^13^C (bottom, 150 MHz) NMR spectra of 5 in THF-d_8_.


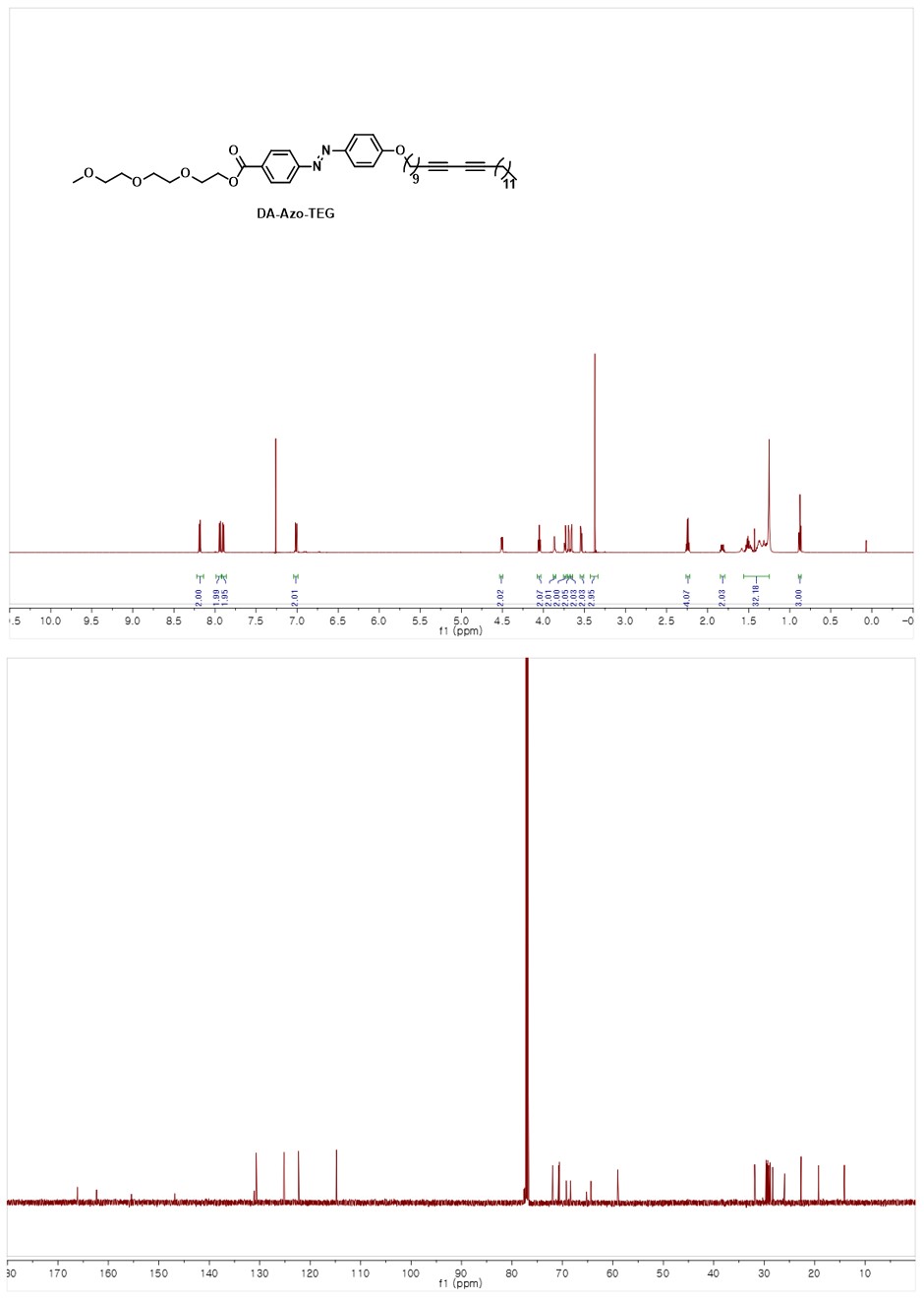


**Supplementary Figure S9.** ^1^H (top, 600 MHz) and ^13^C (bottom, 150 MHz) NMR spectra of **DA-Azo-TEG** in CDCl_3_.

**REFERENCES**

1. Chen, S., Ling, A. & Zhang, H.-L. Synthesis and Phase Behaviors of Side‐Chain Liquid‐Crystalline Polymers Containing Azobenzene Mesogen with the Different Length Alkyl Tail. *J. Polym. Sci. Part A: Polym. Chem.* **51**, 2759-2768 (2013).

2. Mackiewicz, N., Gravel, E., Garaofalakis, A., Ogier, J., John, J., Dupont, D. M., Gombert, K., Tavitian, B., Doris, E. & Ducongé, F. Tumor‐Targeted Polydiacetylene Micelles for In Vivo Imaging and Drug Delivery. *Small* **7**, 2786-2792 (2011).
